# Lidocaine spray 10% prior to intravenous catheterisation in dogs

**DOI:** 10.1186/s13028-022-00639-w

**Published:** 2022-08-20

**Authors:** Emma Hoeberg, Tonje Loken Kolstad, Liisa Martine Moller, Silje Kristine Rosvold, Maren Heggernes Softeland, Henning Andreas Haga, Andreas Lervik

**Affiliations:** Department of Companion Animal Clinical Sciences, Faculty of Veterinary Medicine, Norwegian University of Life Sciences, Oluf Thesens vei 30, 1430 Ås, Norway

**Keywords:** Local anaesthetic, Skin desensitization, Visual analouge scale

## Abstract

A common and to some degree painful procedure in veterinary practice is to insert an intra-venous catheter. In both human and veterinary medicine, a topical mixture of lidocaine and prilocaine (EMLA cream) has shown to reduce the pain, however a period of 60 min between application and initiation of the procedure is recommended. This time lapse is not always suitable for clinical practise and a shorter time before anaesthetic effect is therefore desirable. Lidocaine has a shorter time lapse (1–3 min) when used on mucus membrane; however, the effect of lidocaine for desensitization of skin has shown variable results in humans. The aim of the study was to evaluate the effect of topical lidocaine spray 10% on the response to placement of venous catheters in dogs. Topical lidocaine spray 10% or NaCl 0.9% was administered prior to placing an intravenous catheter in the cephalic vein. A cross-over of treatment with 2 h wash out period was used before placing a catheter in the opposite cephalic vein. The procedure was video recorded and the dogs’ responses were later scored by three persons blinded to treatment using a visual analogue scale. The VAS scores were normalised and the mean difference between treatments were compared using Wilcox signed-rank test. This study could not find a statistical difference between the treatments (P = 0.1763) and could conclude that no significant difference in response to intravenous catheterisation was found between application of NaCl 0.9% or lidocaine 10% prior to the procedure.

## Findings

Placement of intravenous (IV) catheters or venipuncture are common procedures in veterinary practice and known to cause some degree of pain [1]. In both human and veterinary medicine, a topical eutectic mixture of lidocaine and prilocaine (EMLA cream) is described to reduce pain or behavioural response in relation to venipuncture [2–4]. According to the manufacturer, in humans EMLA cream should be applied minimum 60 min prior to the procedure. In dogs similar results were found with less response to catheterisation 60 min compared to 30 min after application of EMLA cream [4]. Lidocaine spray is commonly used to desensitize mucous membranes with an anaesthetic effect after 1–3 min [5]. Lidocaine spray on skin has been evaluated in humans, and lidocaine 8% was shown to reduce filament prick pain [6]. In a paediatric population, lidocaine with adrenaline was more effective compared to EMLA cream in reducing venipuncture pain [7]. However, in adult humans no difference was found in pain evoked by IV catheterization when lidocaine 10% spray was compared to placebo [5]. Based on variable result in human medicine, we wanted to examine the effect of topical application of lidocaine prior to IV catheterisation in dogs. The aim of this study was to evaluate the effect of topical lidocaine 10% applied 5 min before placement of venous catheters in dogs. The null hypothesis was that there is no difference in response to placement of IV catheter after application of NaCl 0.9% compared to lidocaine 10%.

A double-blinded, randomized, balanced, experimental cross-over study was performed. Animals included were privately owned, adult (> 1 year) dogs deemed heathy on clinical examination, without any macroscopically abnormalities at the site for IV catheterization. Dogs with known adverse reaction to lidocaine or behavioural challenges such as aggression or anxiety were excluded. If the dog did not accept restraint or if puncture of the skin failed the dog was excluded. Treatment order as well as first leg to be catheterised were randomised in blocks of four dogs by person A who did not participate in the procedure or evaluation of the response.

The samples size of 12 dogs in study was based on extrapolation from two similar studies in horses and humans, where they based on sample size calculation included respectively 8 and 17 subjects [5, 8].

The dogs were restrained by person B in a sitting position, with the head secured by holding a hand on the head of the dog and turning it away from the person performing the catheterization. The front limb to be catheterised were fixed behind the elbow. An area of 2.5 × 3 cm was clipped over the IV catheterization site mid radius, and the skin disinfected using chlorhexidine with alcohol. The person (A) responsible for randomization applied the treatment. One spray lidocaine 10% (Aspen Pharma Trading Ltd, Dublin, Ireland) corresponding to 0.1 mL or NaCl 0.9% (B. Braun Melsungen AG, Melsungen, Germany) 0.1 mL at a distance of 1 cm from the skin. NaCl was applied using a 1 mL syringe. Five minutes after application IV catheterisation was performed by person C. Catheter size upon discretion of person C. The catheter was removed immediately after placement, and a bandage was placed. The wash out period between catheterizations was minimum 2 h. Prior to the second attempt all dogs went for a 5–10 min walk outside. IV catheterisation was video recorded using two mobile phones. The recording started just prior to the catheterisation attempt and finished after securing the IV catheter. Three persons (C, D, E) blinded to treatment scored the response to IV catheterisation of the two legs separately after all data were collected. A Visual Analogue Scale (VAS) was used, where 0 mm represented no response and 100 mm greatest possible response.

The VAS scores were normalised within each observer as a percentage of the personal maximum score. The mean normalised score for each treatment (mean VAS %), and the difference between lidocaine 10% and NaCl 0.9% was calculated for every observer and dog. Descriptive data are presented as median (range). Data was evaluated visually for normality. For non- normal distributed data paired Wilcoxon signed-rank test was performed and P-value less than 0.05 was considered statistically significant. Statistical analysis was performed using a statistical software JMP 14.1.0 (SAS Institute Inc., Cary, NC, USA).

The study was ethical approved by the National Animal Research Authority number 22132. Participation in the study was voluntary, and all owners signed an informed owner consent form prior to enrolment.

**Fig. 1:**
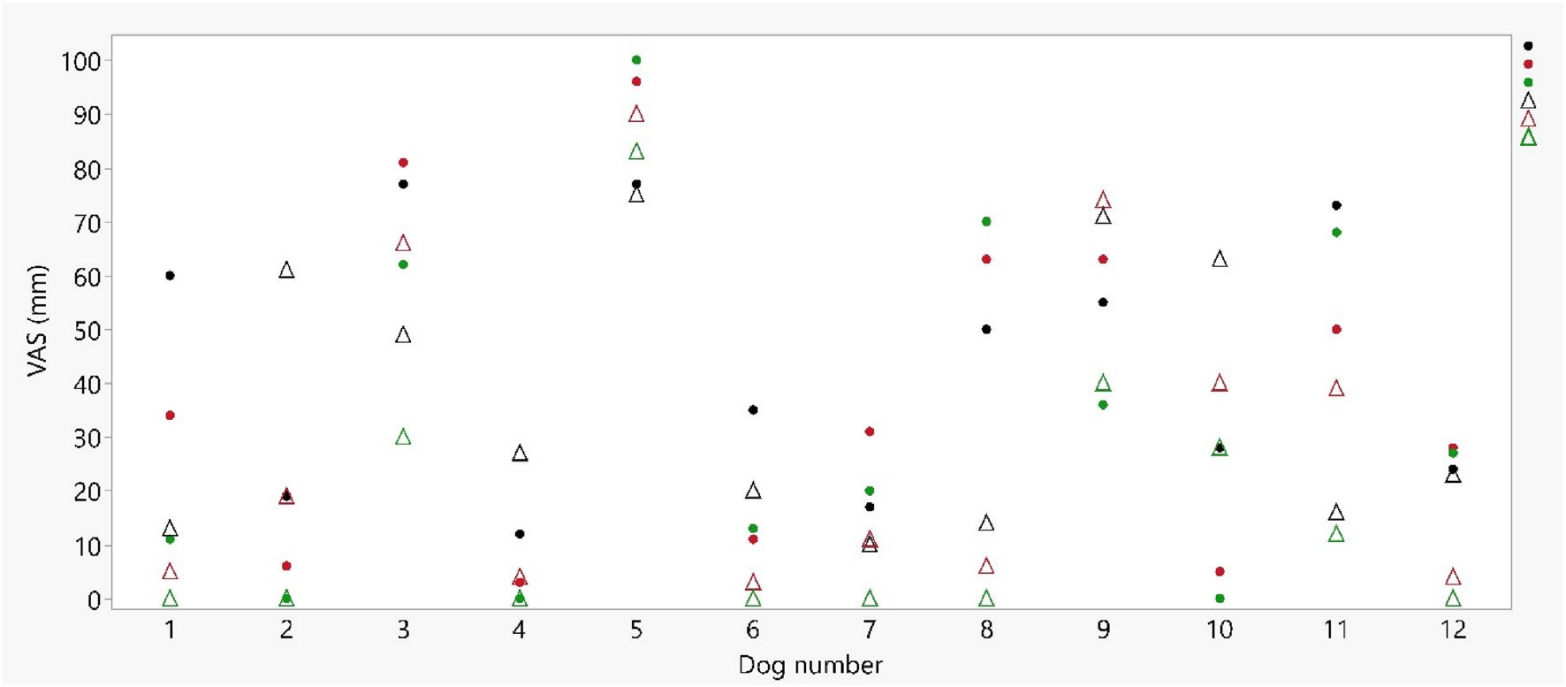
Response to intra-venous catheterization after application of lidocaine spray 10% or NaCl 0.9% in 12 dogs. The response was scored with visual analogue scale (VAS) by three observers. The VAS score in mm is illustrated along the y-axis and along the x-axis each individual dog is illustrated. Each dog has six scores where black represents observer 1, red represents observer 2 and green grey represents observer 3. A circle represents the response after application of lidocaine 10% and a triangle represents the response after application of NaCl 0.9%

**Fig. 2:**
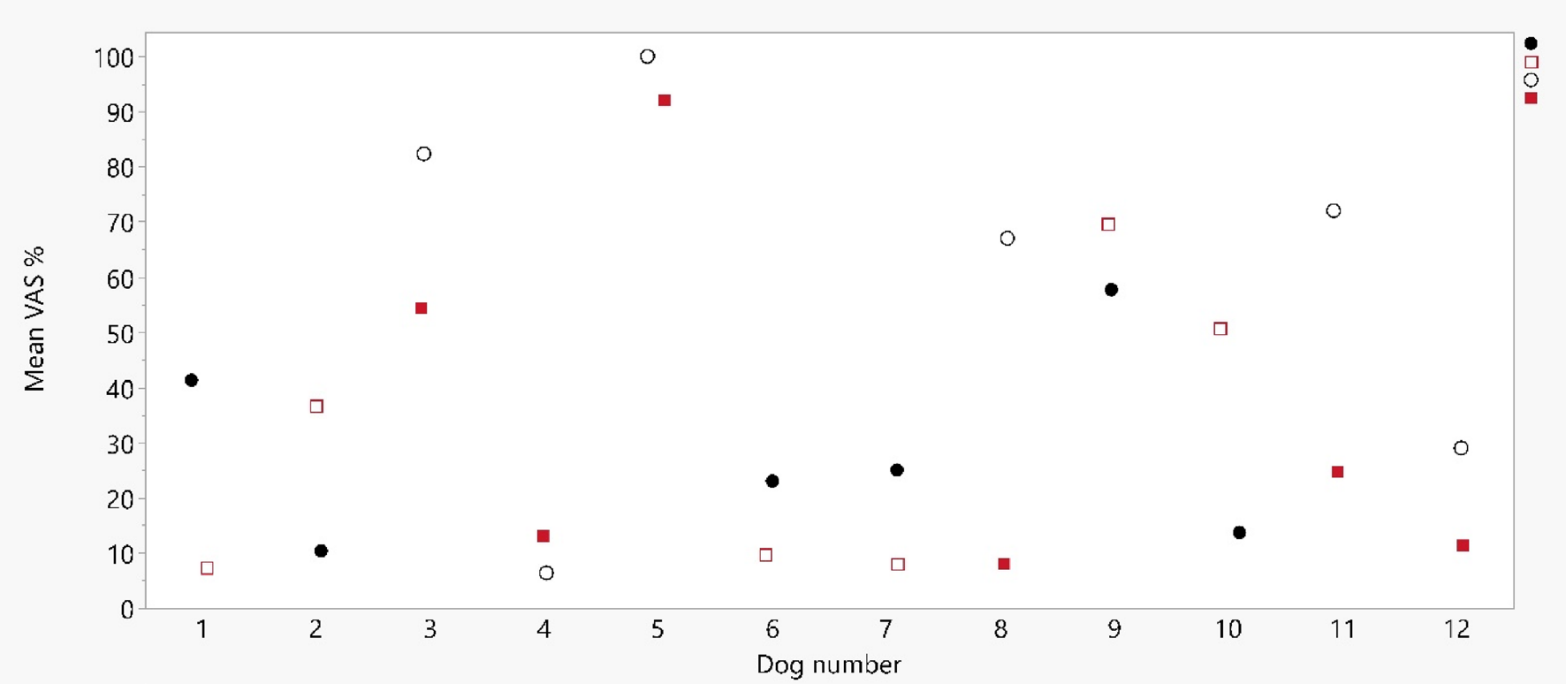
Response to intra-venous catheterization after application of lidocaine spray 10% or NaCl 0.9% in 12 dogs. The response was scored with visual analogue scale by three observers and normalised within each observer. The mean score within each dog was calculated and is illustrated along the y-axis. Along the x-axis each individual dog is illustrated. The response after lidocaine is illustrated by a black circle and for NaCl as a red square, whenever filled, indicates the first treatment

In total, 12 dogs were included in the study with equally distribution of treatment lidocaine 10% and NaCl 0.9% and first limb used. Breeds represented were mixed breed (4), Golden Retriever (2), Cocker Spaniel (2), Labrador Retriever (2), English Setter (1) and Danish-Swedish Farm dog (1) whereby, four intact male and eight intact female dogs, aged 6 (1–12) years with a body mass of 20.9 (4.7–33.9) kg. No dog was excluded due to failure of skin puncture, and an intravenous catheter was successfully placed in 17 of 24 limbs. VAS scores for all observations performed by all observers are presented in Fig. 1. For mean VAS % for each dog and treatment, lidocaine or NaCl, see Fig. 2. Median (interquartile range) was 35.3% (16.2–70.4) and 18.8% (8.5–53.2) in lidocaine and NaCl group, respectively. Paired Wilcox signed- rank test showed no significant difference (P = 0.1763). Eight dogs had a higher mean VAS %-score on the second front limb and four higher on the first front limb. Total mean VAS %-score was increased in the second limb with an increase of 25.6% if NaCl was first treatment and 1.2% if lidocaine was first treatment.

This is the first study reporting topical use of lidocaine spray 10% prior to IV catheterization in dogs. No significant difference in response to IV catheterisation after application of lidocaine 10% or NaCl 0.9% was found. Similar results were found in adult humans when lidocaine spray 10% was applied prior to IV catheterization [5]. However, in other human studies lidocaine was found to have an effect when applied to the skin [6, 7]. An important difference between these studies was the time allowed between application and intervention. When an effect was found, the time between application of lidocaine and intervention was consistently longer, 15 and 13 min respectively [6, 7] compared to the 5 min in the study by Datema et al. [5] and our study. The time lapse of 5 min between application and IV catheterisation in this study might have been too short to fully achieve desensitization of the skin. However, a time lapse of 13–15 min is not always practical in the clinical setting, and the use of 5 min in this study was considered the more reasonable by the investigators. Another possible reason for not detecting a difference between the treatments in our study may have been the lidocaine dose. It is possible that one spray is not enough to cover and desensitize the area prepared for catheterisation. However, in a human study on lidocaine spray 10% prior to IV catheterization, six sprays with a similar volume to our study was used. Despite the higher volume and dose, a difference between treatments could not be found [5]. EMLA cream 5% has been shown to decrease the response to IV catheterization in dogs [4]. The concentration of lidocaine in EMLA cream is lower than that used in our study, it is however a topical eutectic mixture of lidocaine and prilocaine. The formulation and the combination of both prilocaine and lidocaine might be the reason why it decreases the response to IV catheterization at lower concentrations.

A possible limitation of this study was the short wash out period of 2 h between the treatments. The short wash out period between the attempts of IV catheterisation could potentially influence the dogs’ memory and thereby the observed response. We cannot exclude that the experienced discomfort from fixation and IV catheterisation during the first attempt could influence the observed response during the second, regardless of treatment. An interesting finding in our study was that the total mean VAS %-score was increased in the second limb for 60% of the dogs and a larger increase was shown if NaCl 0.9% was first treatment. However, the block randomisation design was considered to ameliorate this effect on comparison between treatments. Twelve dogs were included in the study, and the number of dogs was based on extrapolation from two other studies [5, 8]. In general, extrapolation from other studies should not be used to decide the sample size of a study, but rather a priori power analysis. We performed a post hoc power analysis which revealed that a larger sample size would have been necessary based on the difference found in our study. This mean difference in VAS (%) score is however low, and probably not of clinical relevance. Another limitation was that fully IV catheterisation was not successful in all dogs, however punction of the skin was.

This study could not establish a statistically significant difference in response to placement of IV catheters after application of either NaCl 0.9% or lidocaine 10%. However, further studies with an increased dose and prolonged time between application and catheterisation is desirable.

## Data Availability

The datasets used and/or analysed during the current study are available from the corresponding author on reasonable request.
